# Ferroptosis and HMGB2 induced calreticulin translocation required for immunogenic cell death are controlled by the nuclear exporter XPO1

**DOI:** 10.21203/rs.3.rs-4009459/v2

**Published:** 2024-04-08

**Authors:** Ian Blair, jingqi Fan, Kevin Gillespie, Clementina Mesaros

**Affiliations:** University of Pennsylvania; University of Pennsylvania; University of Pennsylvania; Perelman School of Medicine, University of Pennsylvania

## Abstract

Cisplatin and oxaliplatin cause the secretion of high mobility group box 1 (HMGB1) from cancer cells, which is necessary for initiation of immunogenic cell death (ICD). Calreticulin (CRT) translocation from the endoplasmic reticulum to the plasma membrane is also required; oxaliplatin induces this translocation but cisplatin does not. We have discovered that oxaliplatin causes the secretion of both HMGB1 and HMGB2 from the nucleus into the extracellular milieu. We previously showed that cisplatin mediated secretion of HMGB1 is controlled by the nuclear exporter XPO1 (chromosomal maintenance 1; CRM1). We now find that XPO1 regulates oxaliplatin mediated secretion of both HMGB1 and HMGB2. XPO1 inhibition causes nuclear accumulation of both proteins, inhibition of oxaliplatin-mediated ferroptosis of colon cancer cells, and inhibition of CRT translocation to the plasma membrane of lung and colon cancer cells. Incubation of cancer cells with cell targeted (CT)-HMGB2 confirmed that HMGB2 is responsible for translocation of CRT to the plasma membrane. CT-HMGB2 is three orders of magnitude more potent than oxaliplatin at inducing CRT translocation. Inhibition of HMGB1 and HMGB2 secretion and/or their activation of nuclear factor-kappa B (NF-kB) has potential utility for treating cardiovascular, and neurodegenerative diseases; whereas CT-HMGB2 could augment therapeutic approaches to cancer treatment.

## Introduction

**Figure 1:**
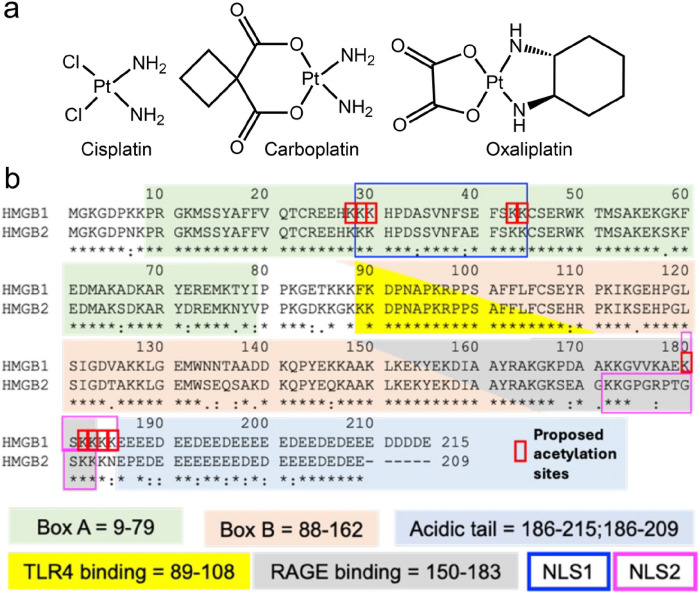
Structures of platinum drugs and amino acid sequences of HMGB proteins. (**a**) Chemical structures of cisplatin, carboplatin, and oxaliplatin. (**b**) Alignment of amino acid sequences of HMGB1 (upper, 215 amino acids) and HMGB2 (lower, 209 amino acids). HMGB1 has a longer acidic tail and a different NLS2 when compared with HMGB2. The TLR4 binding regions (89–108) are identical and NLS1 regions differ only in A-34 and S-39 being transposed; whereas the RAGE binding regions (150–183) are only 79 % identical.

The first-generation platinum drug, cisplatin, and the second-generation platinum drug, oxaliplatin^1^ both induce the secretion of similar amounts of high mobility group box 1 (HMGB1) protein from cancer cells (Fig. 1a)^2,3^. In contrast, another second-generation platinum drug, carboplatin^1^ (Fig. 1a) does not^3^. Secretion of HMGB1 is necessary for the induction of immunogenic cell death (ICD)^4^. The concept of ICD was first introduced by Casares *et al*. in 2005 as a term to explain how tumor cells dying after treatment with anthracyclines can elicit an effective immune response, which causes the suppression of tumor growth.^5^ Oxaliplatin but not cisplatin induces ICD even though cisplatin induces similar amounts of HMGB1 secretion, implying that there is an additional unidentified mechanism of ICD^2^. Both HMGB1 (also known as amphoterin) and HMGB2 are abundant non-histone nuclear proteins. HMGB1 is 93 % homologous and 80 % identical to HMGB2 (Fig. 1b)^6^. HMGB1 has been studied much more extensively than HMGB2 with 17-fold more publications, and consequently its biological functions are better delineated including its role as a danger-associated molecular pattern (DAMP)^7^. Both HMGB1 and HMGB2 contain three conserved regions that are structurally related^7^. The A box at the amino terminus is a DNA-binding region, which binds to AT-rich sequences of DNA; whereas the B box binds to selected DNA sequences and bends them to alter their structures (Fig. 1b)^7–9^. A third region comprises a long acidic tail, which in HMGB1 includes 20 glutamate and 10 aspartate residues (Fig. 1b). The acidic tail of HMGB2 is a little shorter with 18 glutamate and only 5 aspartate residues as well as a single non-polar proline residue (Fig. 1b).

It has been reported that HMGB1 can be secreted into the circulation in numerous diseases including neurodegeneration^10^, Alzheimer disease^11^, lung disease^12^, and a wide range of cancers including gastric cancer^13^, colorectal cancer^13,14^, hepatocellular carcinoma^13^, pancreatic cancer^13,15^, nasopharyngeal carcinoma^13^, head and neck squamous-cell carcinoma^13,16^, esophageal cancer^13^, malignant pleural mesothelioma^13^, bladder cancer^13^, prostate cancer^13,17^, ovarian cancer^17^, lung cancer^18^, and cervical carcinoma^13^. Fewer studies have examined the secretion of HMGB2^19^, although it was recently discovered that nuclear HMGB2 makes an important contribution to the differentiation and survival of functional memory cells and stem-like progenitor exhausted T cells^20^. Previous studies had shown that HMGB2 is also involved in the differentiation of stem cells during spermatogenesis^21^, neural stem cell development^22^, and myogenesis^23^.

HMGB2 can be mistaken for secreted HMGB1 when using enzyme-linked immunosorbent assays (ELISAs) or western blot analysis because many antibodies cannot distinguish the two proteins^24^. Therefore, we explored the possibility that HMGB2 (in addition to HMGB1) might be secreted by cancer cells in response to oxaliplatin, explaining the difference in its induction of ICD when compared with cisplatin. Previously, we definitively showed that cisplatin does not induce the secretion of HMGB2 from cancer cells^3^. This was accomplished using a highly specific and sensitive method based on immunoprecipitation (IP) stable isotope dilution nano-liquid chromatography-parallel reaction monitoring/high resolution mass spectrometry (nano-LC-PRM/HRMS). The method was also used to show that oxidized HMGB1 proteoforms (oxidized cysteine residues) are the major molecular species secreted from hepatocytes after exposure to high concentrations of acetaminophen^25^.

A modification of the original nano LC-PRM/HRMS method for HMGB1^6^ was applied to the analysis of HMGB proteoforms that are secreted from human non-small cell lung cancer (NSCLC) and colon cancer cells in response to oxaliplatin. In addition, western blot analysis using highly specific anti-HMGB1 and anti-HMGB2 antibodies was conducted. We also conducted mechanistic studies to determine whether oxaliplatin mediated HMGB proteoform secretion from human NSCLC and colon cancer cells is also mediated by the nuclear exporter XPO1 and how this relates to ICD.

## Results

### A549 NSCLC cells secrete both HMGB1 and HMGB2 in response to oxaliplatin

**Figure 2:**
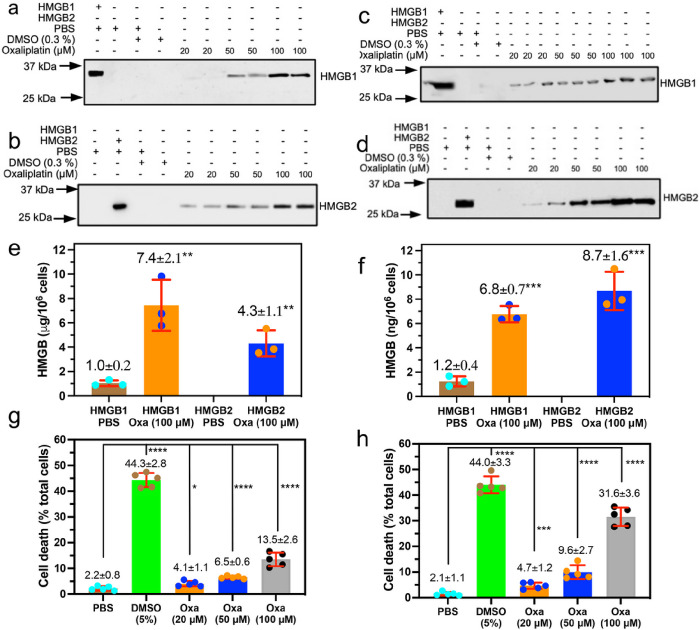
Increased secretion of HMGB1 and HMGB2 from human cancer cells and increased cell death is caused by increasing amounts of oxaliplatin. HMGB secretion after incubations with PBS (control), 0.5 % DMSO (control), or with 20 mM, 50 mM, or 100 mM oxaliplatin for 24-h. (**a**) HMGB1 secreted from A549 cells. (**b**) HMGB2 secreted from A549 cells. (**c**) HMGB1 secreted from HCT116 cells (**d**) HMGB2 secreted from HCT116 cells. Western blots are shown in the upper panels and Image J^69^ quantification of relative intensities of the blots are shown in the lower panels. Nano LC-PRM/MS absolute quantification of HMGB1 and HMGB2 secreted by PBS or 100 mM oxaliplatin for 24-h. (**e**) A549 cells. (**f**). HCT116 cells. Dead cell count as a % of total cell count after incubations with PBS (control), 5 % DMSO (positive control), or with 20 mM, 50 mM, or 100 mM oxaliplatin. (**g**) A549 cells (**h**) HCT116 cells. Western blot data are from two or three biological replicates. Mass spectrometry data are from three biological replicates and cell viability assays are from five biological replicates; *p < 0.01, **p < 0.008, ***p < 001, ****p < 0.0001.

We have now determined that oxaliplatin (Fig. 1a) can secrete both HMGB1 and HMGB2. Importantly, the rabbit polyclonal antibody (pAb) used to detect HMGB1, which was raised against the C-terminal acidic tail of HMGB1, was able to distinguish HMGB1 from HMGB2 by western blot analysis (Fig. 2a, upper; lanes 2 and 3). In addition, the rabbit pAb used to detect HMGB2, which was raised against an N-terminal HMGB2 peptide of unspecified sequence, was able to distinguish HMGB2 from HMGB1 by western blot analysis (Fig. 2b, upper; lanes 2 and 3). Anti-HMGB immunoblots of cell culture media revealed that both HMGB1 (Fig. 2a, upper) and HMGB2 (Fig. 2b, upper) were secreted by A549 NSCLC cells incubated with oxaliplatin in a dose-dependent manner. Secreted HMGB1 appeared at a mobility corresponding to a molecular weight (MW) of 27 kDa, which was close to the mobility of a His-tagged authentic standard of HMGB2 (calculated MW = 25,717 Da; Fig. 2a, upper; lane 2). HMGB2 ran slightly faster, appearing at a mobility corresponding to a MW of 25 kDa, which was close to the mobility of a His-tagged authentic standard of HMGB2 (calculated MW = 24,857; Fig. 2b, upper; lane 3). Control experiments with PBS and 0.5 % dimethyl sulfoxide (DMSO) showed that there was very little secretion of either of the two HMGB proteins (Figs. 2a upper and 2b upper, lanes 4 and 5). Quantification of the relative intensities confirmed that increasing amounts of oxaliplatin up to 100 mM caused the secretion of increasing amounts of HMGB1 (Fig. 2a, lower) and HMGB2 (Fig. 2b, lower) into the A549 cell media. Absolute quantification was conducted by stable isotope dilution nano LC-PRM/HRMS analysis of the HMGB1 and HMGB2 secreted into the A549 NSCLC cell media after incubation with 100 mM oxaliplatin for 24-h (Fig. 2e). The amount of HMGB1 present in the A549 cell media increased from 1.0 ± 0.2 mg/10^6^ cells or 5.8 ± 1.2 nM in the PBS controls (n = 3) to 7.4 ± 2.1 mg/10^6^ cells or 43.2 ± 12.2 nM (n = 3) after a 24- h incubation with 100 mM oxaliplatin (p = 0.006). The amount of HMGB2 present in the A549 cell media increased from below the limit of quantification in the PBS controls (n = 3) to 4.3 ± 1.1 mg/10^6^ cells or 25.1 ± 7.0 nM (n = 3) after a 24-h incubation with 100 mM oxaliplatin (p = 0.002).

### HCT116 colon cancer cells secrete both HMGB1 and HMGB2 in response to oxaliplatin

Having established that NSCLC cells secreted both HMGB1 and HMGB2 in response to oxaliplatin, we next established whether colon cancer cells could also secrete both HMGBs. Anti-HMGB1 immunoblots of HCT116 cell culture media revealed that both HMGB1 (Fig. 2c, upper) and HMGB2 (Fig. 2d, upper) were secreted after oxaliplatin treatment in a dose-dependent manner. The HMGB1 (Fig. 2c, upper; lane 2) and HMGB2 (Fig. 2d, upper; lane 3) appeared at mobilities close to those observed for A549 cell media (Figs. 2a upper and 2b upper, lanes 2 and 3). Control experiments with PBS and 0.5 % DMSO again revealed very little secretion of either of the two HMGB proteins (Figs. 2c upper and 2d upper, lanes 4 and 5). Quantification of the relative intensities confirmed that increasing amounts of oxaliplatin up to 100 mM caused the secretion of increasing amounts of HMGB1 (Fig. 2c, lower) and HMGB2 (Fig. 2d, lower) into the HCT116 media. Absolute quantification was conducted by stable isotope dilution nano LC-PRM/HRMS analysis of the HMGB1 and HMGB2 secreted into the HCT116 cell media after incubation with 100 mM oxaliplatin for 24-h (Fig. 2f). The amount of HMGB1 present in the HCT116 cell media increased from 1.2 ± 0.4 mg/10^6^ cells or 7.0 ± 2.3 nM in the PBS controls (n = 3) to 6.8 ± 0.7 mg/10^6^ cells or 39.7 ± 4.1 nM (n = 3) after a 24-h incubation with 100 mM oxaliplatin (p = 0.0003). The amount of HMGB2 present in the HCT116 cell media increased from below the limit of quantification in the PBS controls (n = 3) to 8.7 ± 1.6 mg/10^6^ cells or 50.7 ± 9.3 nM (n = 3) after a 24-h incubation with 100 mM oxaliplatin (p = 0.0007).

### Increase of cell death with increasing doses of oxaliplatin

Having determined that concentrations of oxaliplatin up to 100 mM could induce both cell lines to secrete HMGB1 as well as HMGB2, we next established whether these concentrations of oxaliplatin could also reduce cell viability and induce cell death. A549 NSCLC cell death increased to 4.1 ± 1.1 % (n = 5) with 20 mM oxaliplatin when compared with PBS controls (2.2 ± 0.8 %, n = 5, p = 0.001; Fig. 2g). Cell death increased further to 6.5 ± 0.6 % (p = 0.0001, n = 5; Fig. 2g) with 50 mM oxaliplatin and to 13.5 ± 2.6 % (p < 0.0001, n = 5; Fig. 2g) with 100 mM oxaliplatin. Similarly, HCT116 colon cancer cell death increased to 4.7 ± 1.2 % (n = 5) with 20 mM oxaliplatin when compared with PBS controls (2.1 ± 1.1 %, n = 5, p = 0.001; Fig. 2h). Cell death increased further to 9.6 ± 2.7 % (p = 0.0001, n = 5; Fig. 2h) with 50 mM oxaliplatin and to 31.6 ± 3.6 % (p < 0.0001, n = 5; Fig. 2h) with 100 mM oxaliplatin.

### Nuclear HMGB1 and HMGB2 in cancer cells is reduced by treatment with oxaliplatin

**Figure 3:**
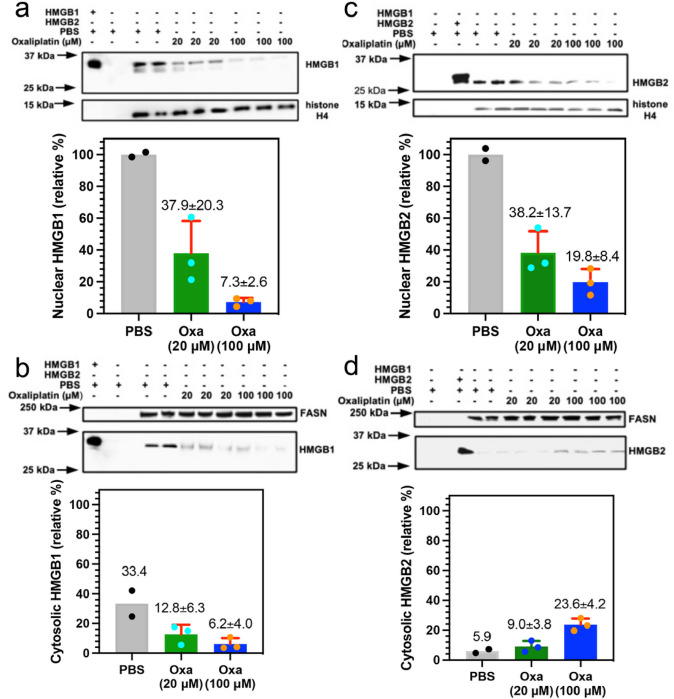
Sub-cellular localizations of HMGB1 and HMGB2 in A549 NSCLC cells change after treatment with oxaliplatin. HMGB analysis after incubations with 20 mM or 100 mM oxaliplatin for 24-h. (**a**) HMGB1 in the nucleus. (**b**) HMGB1 in the cytosol. (**c**) HMGB2 in the nucleus. (**d**) HMGB2 in the cytosol. Western blots are shown in the upper panels and Image J^69^ quantification of blots in the lower panels. Histone H4 and FASN were used as loading controls for nuclear and cytosolic protein respectively. Cytosolic HMGB1 and HMGB2 were compared with HMGB1 or HMGB2 in the nucleus of the relevant PBS treated cells. Western blot data are from three biological replicates.

**Figure 4:**
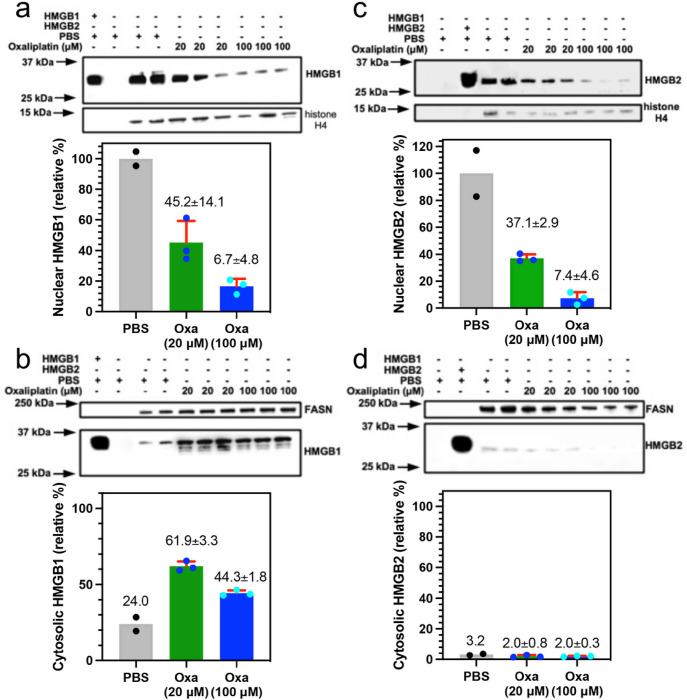
Sub-cellular localizations of HMGB1 and HMGB2 in HCT116 cells change after treatment with oxaliplatin. HMGB analysis after incubations with 20 mM or 100 mM oxaliplatin for 24-h. (**a**) HMGB1 in the nucleus. (**b**) HMGB1 in the cytosol. (**c**) HMGB2 expressed in the nucleus. (**d**) HMGB2 expressed in the cytosol. Western blots are shown in the upper panels and Image J^69^ quantification of blots in the lower panels. Histone H4 and FASN were used as loading controls for nuclear and cytosolic protein respectively. Cytosolic HMGB1 and HMGB2 were compared with HMGB1 or HMGB2 in the nucleus of the relevant PBS treated cells. Western blot data are from three biological replicates.

The mechanism of HMGB protein secretion was examined in more detail by analyzing HMGB1 and HMGB2 in the nucleus and cytosol after incubating the A549 NSCLC and HCT116 colon cancer cells with oxaliplatin. HMGB1 was present in the nucleus (Fig. 3a) and cytosol (Fig. 3b) of the A549 NSCLC cell PBS controls; whereas HMGB2 was only present the nucleus (Fig. 3c) and was close to the detection limit in the cytosol (Fig. 3d). There was a significant reduction in HMGB1 in the nucleus to 37.9 ± 20.3 % (Fig. 3a) and cytosol to 12.8 ± 6.3 % (n = 3, Fig. 3b) after incubation with 20 mM oxaliplatin for 24-h and it was reduced still further in the nucleus to 7.3 ± 2.6 % (n = 3, Fig. 3a) and cytosol t 6.2 ± 24.0 % (n = 3 Fig. 3b) after incubation with 100 mM oxaliplatin for 24-h. In contrast, there was a similar significant decrease in HMGB2 in the nucleus to 38.2 ± 13.7 % n = 3. (Fig. 3c), but it was increased in the cytosol from barely being detected to 9.0 ± 3.8 % (n = 3, Fig. 3d) after incubation with 20 mM oxaliplatin for 24-h. HMGB2 was reduced still further in the nucleus to 19.8 ± 8.4 % (n = 3, Fig. 3c) after incubation with 100 mM oxaliplatin for 24-h but increased substantially in the cytosol to 23.6 ± 4.2 % (n = 3. Fig. 3d). Similarly, HMGB1 was present in the nucleus (Fig. 4a) and cytosol (Fig. 4b) of the HCT116 lung cancer cell PBS controls; whereas HMGB2 was present the nucleus (Fig. 4c) but could barely be detected in the cytosol (Fig. 4d). There was a reduction in HMGB1 in the nucleus of HCT116 cells after incubation with 20 mM oxaliplatin for 24-h to 45.2 ± 14.1 % (n = 3) and it was reduced still further to 6.7 ± 4.8 % (n = 3) after incubation with 100 mM oxaliplatin for 24-h (Fig. 4a). In contrast, HMGB1 in the cytosol increased substantially to 61.9 ± 3.3 % (n=3) after incubation with 20 mM oxaliplatin for 24-h and remained elevated at 44.3 ± 1.8 % (n = 3) after incubation with 100 mM oxaliplatin for 24-h (Fig. 4b). There was also a major reduction of HMGB2 in the nucleus to 37.1 ± 2.9 % (n = 3) after incubation with 20 mM oxaliplatin for 24-h and it was reduced still further to 7.4 ± 4.6 % (n = 3) after incubation with 100 mM oxaliplatin for 24-h (Fig. 4c). In contrast to the A549 cells, HMGB2 was present at very low levels in the cytosol of HCT116 cells after incubation with 20 mM oxaliplatin at 2.0 ± 0.8 % (n = 3, Fig. 4d) or 100 mM oxaliplatin at 2.0 ± 0.3 % after 24-h (n = 3, Fig. 4d). These results revealed that oxaliplatin caused nuclear HMGB1 and HMGB2 secretion from both A549 NSCLC cells and HCT116 colon cancer cells.

### Oxaliplatin induced HMGB1 and HMGB2 secretion is mediated by the nuclear exporter XP01

**Figure 5:**
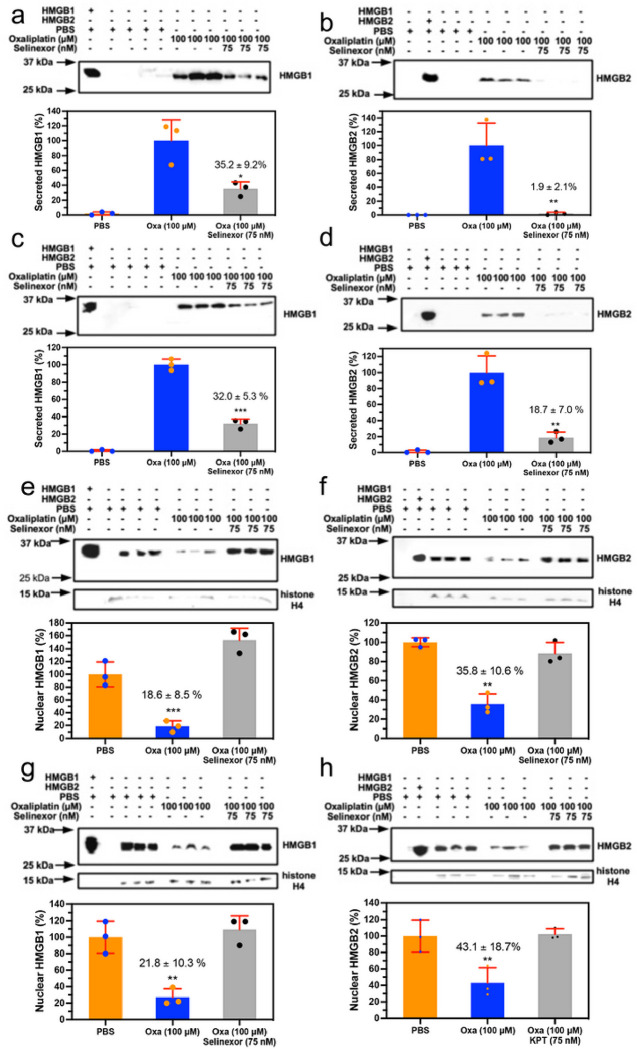
Extracellular HMGB1 and HMGB2 excretion from the nucleus is inhibited by Selinexor an inhibitor of the nuclear exporter XPO1. HMGB analysis after incubations with PBS buffer (control), 100 mM oxaliplatin, or 100 mM oxaliplatin + 75 nM Selinexor. (**a**) HMGB1 in A549 cell media. (**b**) HMGB2 in A549 cell media (**c**) HMGB1 in HCT116 cell media. (**d**) HMGB2 in HCT116 cell media. (**e**) HMGB1 in A549 cell nucleus. (**f**) HMGB2 in A549 cell nucleus (**g**) HMGB1 in HCT116 cell nucleus. (**h**) HMGB2 in HCT116 cell nucleus. Western blots are shown in the upper panels and Image J^69^ quantification of blots in the lower panels. Western blot data are from three biological replicates. Histone H4 was a loading control for the nuclear fraction; *p < 0.05, **p < 0.01, ***p < 0.001.

We previously showed that inhibition of XP01 with 75 nM Selinexor caused a reduction in cisplatin induced HMGB1 secretion^3^. This suggested that Selinexor might also reduce oxaliplatin induced secretion of both HMGB1 and HMGB2. We found that there was a significant reduction in HMGB1 secretion to 35.2 ± 9.2% (n = 3, p = 0.0193; Fig. 5a) and HMGB2 secretion to 1.9 ± 2.1 % (n = 3, p = 0.0065; Fig. 5b) from A549 NSCLC cells incubated with 100 mM oxaliplatin when 75 nM Selinexor was added. Similarly, there was a significant reduction of HMGB1 to 32.0 ± 5.3% (n = 3, p = 0.0001; Fig. 5c) and HMGB2 to 18.7 ± 7.0 % (n = 3. p = 0.0030; Fig. 5d) secreted by HCT116 colon cancer cells incubated with 100 mM oxaliplatin when 75 nM Selinexor was added. Incubation of Selinexor (75 nM) alone with A549 NSCLC cells or HCT116 colon cancer cells had no effect on HMGB1 or HMGB2 secretion (data not shown).

### Inhibition of nuclear export reverses the oxaliplatin induced loss of nuclear HMGB1 and HMGB2

There was no significant difference in HMGB1 (n = 3, 153.8 ± 9.2%; Fig. 5e) or HMGB2 (n = 3, 88.5 ± 11.5 %; Fig. 5f) in the nucleus of A549 NSCLC cells when compared with PBS controls after the addition of 100 mM oxaliplatin + 75 nM Selinexor. This contrasted with the result from the addition of 100 mM oxaliplatin alone to A549 cells where HMGB1 was significantly reduced to 18.6 ± 2.1 % (n = 3, p = 0.0003; Fig. 5e) and HMGB2 was significantly reduced to 3.8 ± 2.1 % (n = 3, p = 0.004; Fig. 5f) when compared with PBS controls. Similarly, there was no significant difference in HMGB1 (n = 3, 109.3 ± 16.7 %; Fig. 5g) or HMGB2 (n = 3 102.2 ± 6.4 %; Fig. 5h) in the nucleus of HCT116 cells when compared with PBS controls after the addition of 100 mM oxaliplatin + 75 nM Selinexor. Again, this contrasted with the result from the addition of 100 mM oxaliplatin alone to HCT116 cells where HMGB1 was significantly reduced to 21.8 ± 10.3 % (n = 3, p = 0.002; Fig. 5g) and HMGB2 was significantly reduced to 43.1 ± 18.7 (n = 3, p = 0.006; Fig. 5h) when compared with PBS controls.

### Inhibition of nuclear export of HMGB1 and HMGB2 reverses oxaliplatin induced translocation of calreticulin (CRT) from the cytosol to the plasma membrane

**Figure 6:**
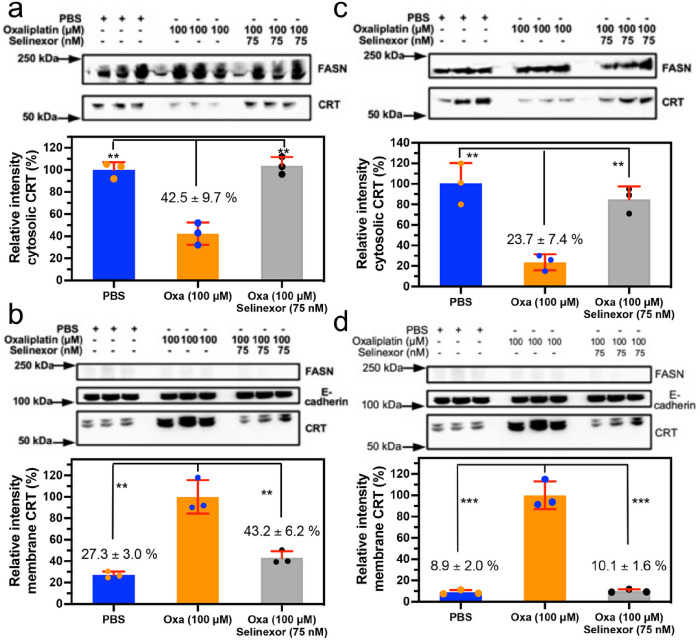
Oxaliplatin-induced CRT translocation from the cytosol to the plasma membrane is inhibited by Selinexor. Analysis of CRT in the cytosol and on the plasma membrane after incubations with PBS buffer (control), 100 mM oxaliplatin, or 100 mM oxaliplatin + 75 nM Selinexor for 24-h. (**a**) Cytosol of A549 cells. (**b**) Plasma membrane of A549 cells. (**c**) Cytosol of HCT116 cells. (**d**) Plasma membrane of HCT116 cells. Western blots are shown in the upper panels and Image J^69^ quantification of blots in the lower panels. FASN and E-cadherin were used as loading controls for cytosolic proteins and membrane proteins, respectively. Western blot data are from three biological replicates; **p < 0.01, ***p <0.001.

The ability to inhibit HMGB1 and HMGB2 secretion from the nucleus into the cytosol made it possible to examine the consequences of this inhibition on the translocation of CRT from the endoplasmic reticulum to the plasma membrane, a process that is critical for initiating of ICD^2,26,27^. Incubation of A549 NSCLC cells with oxaliplatin alone caused a significant reduction of cytosolic CRT to 42.5 ± 9.7 % (n = 3) of the PBS controls (p = 0.001; Fig. 6a). This reduction was reversed by the addition of 75 nM Selinexor, where cytosolic CRT levels of 103.7 ± 8.0 % (n = 3) were similar to PBS controls but significantly different from oxaliplatin alone (n = 3, p = 0.001, Fig. 6a). In contrast, the levels of plasma membrane CRT in PBS controls were 27.2 ± 3.0 % (n = 3) of the cells that were incubated with oxaliplatin alone (p = 0.001; Fig. 6b). This reduction was reversed by the addition of 75 nM Selinexor, where the plasma membrane CRT levels of 43.2 ± 6.2 % (n=3) were similar to PBS controls but significantly different from oxaliplatin alone (n = 3, p = 0.004, Fig. 6b). Similarly, incubation of HCT116 colon cancer cells with oxaliplatin alone caused a significant reduction of cytosolic CRT to 23.7 ± 7.4 % (n = 3) of the PBS controls (p = 0.004, Fig. 6c). Again, this reduction was reversed by the addition of 75 nM Selinexor, where cytosolic CRT levels were similar to PBS controls (Fig. 6c) but significantly different from oxaliplatin alone (n=3, p = 0.002, Fig. 6c). In contrast, the levels of plasma membrane CRT in PBS controls were 8.9 ± 2.0 % (n = 3) of the levels in HCT116 cells that were incubated with oxaliplatin alone (p = 0.0003; Fig. 6d). This reduction was reversed by the addition of 75 nM Selinexor, where the plasma membrane CRT levels of 10.1 ± 1.6 % (n = 3) were similar to PBS controls (Fig. 6d), different from oxaliplatin alone (n=3, p = 0.003, Fig. 6d).

### Inhibition of oxaliplatin induced CRT translocation from the cytosol to the plasma membrane by Selinexor can be visualized by immunofluorescence and flow cytometry

**Figure 7:**
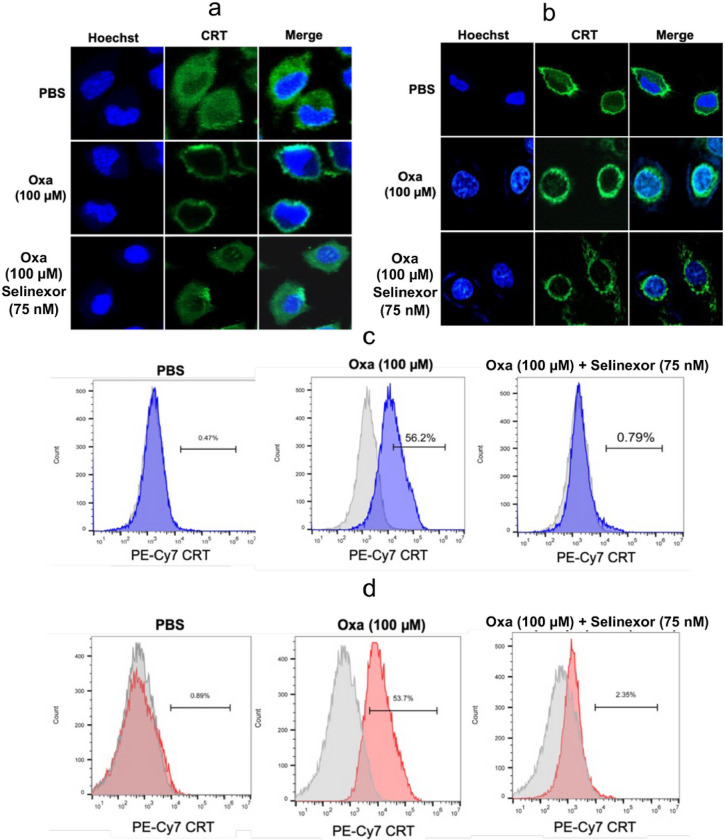
Oxaliplatin-induced CRT translocation from the cytosol to the plasma membrane surface is inhibited by Selinexor. Hoechst immunofluorescent blue signals from nuclear DNA and immunofluorescent green signals from CRT in cells together with merged signals after incubations with PBS buffer (control), 100 mM oxaliplatin, or 100 mM oxaliplatin + 75 nM Selinexor for 24-h. (**a**) A549 cells. (**b**) HCT116 cells. FACS analysis of cells with no fluorescent labeling (gray) or cells with a CRT fluorescent tag on the plasma membrane surface (blue or red) after incubating the cells with PBS buffer (control), 100 mM oxaliplatin, or 100 mM oxaliplatin + 75 nM Selinexor for 24-h. (**c**) A549 cells with a CRT fluorescent tag on the plasma membrane surface (shown in blue). (**d**) HCT116 cells with a CRT fluorescent tag on the plasma membrane surface (shown in red). Representative results are shown from single experiments that were conducted on two different days.

Immunofluorescence and flow cytometry was used to confirm that CRT was located specifically on the plasma membrane surface. CRT permeabilized in A549 or HCT116 cells fixed with paraformaldehyde was visualized with a primary mouse pAb to CRT and fluorescent labeled secondary goat anti-mouse pAb with excitation at 490 nm and emission at 525 nm. The green fluorescence signal for CRT in A549 cells was localized to the cytosolic compartment with minimal signal on the plasma membrane surface (Fig. 7a). Merging the blue Hoechst and green CRT signals showed that no CRT had translocated to the nucleus (Fig. 7a). After a 24 h incubation with oxaliplatin, the green fluorescence was observed primarily on the plasma membrane surface (Fig. 7a). Again, merging the blue Hoechst and green CRT signals showed that no CRT had translocated to the nucleus (Fig. 7a). In contrast, the addition of 75 nM Selinexor to the oxaliplatin incubation provided fluorescent images that were very similar to those obtained with the PBS control (Fig. 7a). Essentially identical images were obtained with the HCT 116 colon cancer cell line (Fig. 7b). Flow cytometry was also used to determine if the CRT was on the plasma membrane surface of the two cell lines. A549 or HCT116 cells were first pre-incubated with a mouse pAb that recognized CRT on the cell surface. After the incubation of A549 cells with oxaliplatin for 24-h, 56.2 % of the cells had CRT on their plasma membrane cell surface (Fig. 7c); whereas only 0.4 % of cells incubated with PBS had CRT on their cell surface (Fig. 7c). Addition of 75 nM Selinexor to the oxaliplatin almost completely prevented the CRT translocation so that only 0.8 % of the cells had CRT on their plasma membrane surface (Fig. 7c). Essentially identical results were obtained for HCT116 colon cancer cells (Fig. 7d).

### Low doses of cell targeted (CT)-HMGB2 can induce the translocation of CRT from the cytosol to the plasma membrane surface

**Figure 8:**
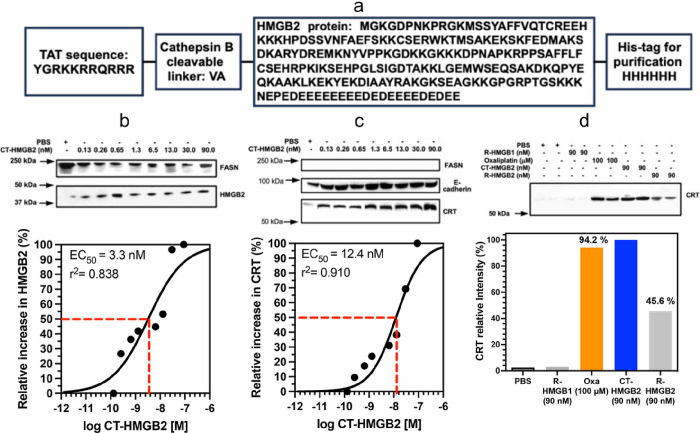
CT-HMGB2 induces translocation of CRT to the plasma membrane surface of A549 cells. (**a**) Amino acid sequence of CT-HMGB2 showing the TAT sequence and the VA linker that is cleaved by cytosolic cathepsin B. (**b**) Western blots of HMGB2 in the cytosol of A549 cells after incubations with CT-HMGB2 from 0.13 nM to 90 nM for 24-h with Image J quantification^69^ of the relative amounts (EC_50_ = 3.3 nM). (**c**) Western blots of CRT on the plasma membrane of A549 cells after incubations with PBS (control) or increasing amounts of CT-HMGB2 from 0.13 nM to 90 nM for 24-h with Image J quantification of the relative amounts (EC_50_ = 12.4 nM). (**d**) Western blots of CRT isolated by IP of A549 cell membranes showing mean relative amounts compared to CT-HMGB2 after duplicate incubations with PBS (control), R-HMGB1 (90 nM), oxaliplatin (100 μM), CT-HMGB2 (90 nM), R-HMGB2 (90 nM) for 24-h. E-cadherin and FASN were used as a loading control for membrane proteins and to reveal cytosolic contamination, respectively.

**Figure 9:**
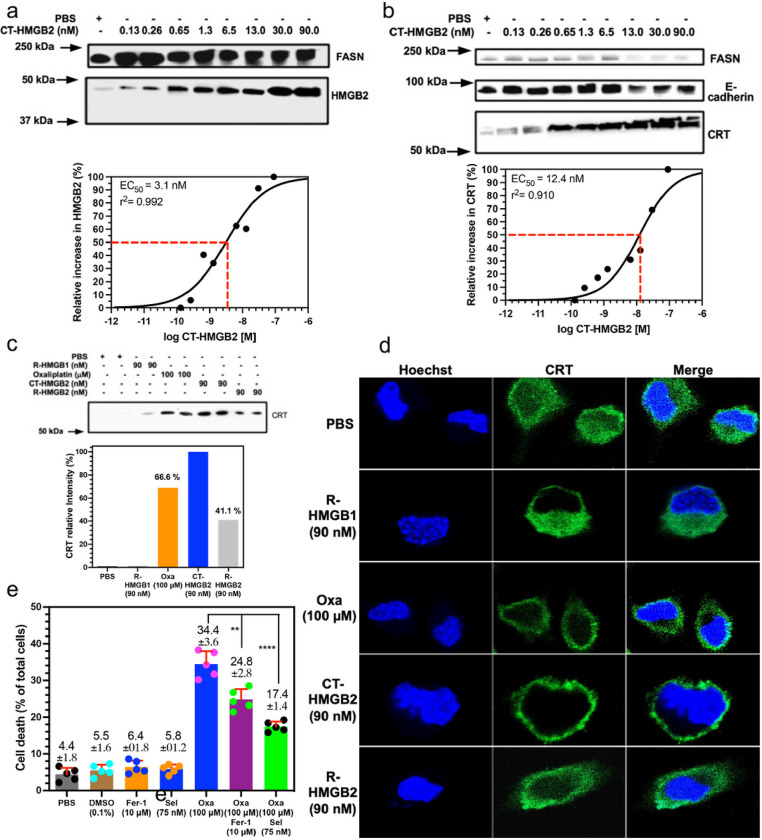
CT-HMGB2 induces translocation of CRT to the plasma membrane surface of HCT116 cells and oxaliplatin-mediated ferroptosis of HCT116 cells is reduced by Selinexor. (**a**) Western blots of HMGB2 in HCT116 cell cytosol after incubations with CT-HMGB2 from 0.13 nM to 90 nM for 24-h with Image J quantification^69^ of the relative amounts (EC_50_ = 3.1 nM). (**b**) Western blots of CRT on the plasma membrane of HCT116 cells after incubations with PBS (control) or increasing amounts of CT-HMGB2 from 0.13 nM to 90 nM for 24-h with Image J quantification of the relative amounts (EC_50_ = 12.4 nM). (**c**) Western blots of CRT isolated by IP from HCT116 cell membranes showing mean relative amounts compared to CT-HMGB2 after duplicate incubations with PBS (control), R-HMGB1 (90 nM), oxaliplatin (100 μM), CT-HMGB2 (90 nM), R-HMGB2 (90 nM) for 24-h. **(d)** Hoechst immunofluorescent blue signals from nuclear DNA and immunofluorescent green signals from CRT in HCT116 cells together with merged signals after incubations with PBS, R-HMGB1 (90 nM), R-HMGB2 (90 nM), oxaliplatin (100 mM), or CT-HMGB2 (90 nM) for 24-h. (**e**) Dead cells as a % of total cells after incubations with PBS (control), 0.1 % DMSO (control), Selinexor (75 nM), oxaliplatin (100 mM), oxaliplatin (100 mM) + ferrostatin-1 (10 mM), or oxaliplatin (100 mM) + Selinexor (75 nM) for 24-h. E-cadherin and FASN were used as a loading control for membrane proteins and to reveal possible cytosolic contamination, respectively. Cell viability assays were conducted on five biological replicates; **p< 0.01, ****p <0.0001.

To test whether secretion of HMGB2 into the cytosol could cause CRT translocation, cell targeted HMGB2 (CT-HMGB2, Fig. 8a) was incubated with the NSCLC and colon cancer cell lines in increasing doses from 0.13 nM to 90 nM for 24-h. The twin arginine targeting (TAT) sequence (YGRKKRRQRRR)^28^ at the N-terminus of CT-HMGB2 (Fig. 8a) would facilitate transport across the plasma membrane and that intracellular cleavage of the TAT sequence by cathepsin B^29^ at the VA linker would then occur to release cytosolic HMGB2. After 24-h, CT-HMGB2 could not be detected in the cell media of A549 cells or HCT116. This was confirmed by the dose-dependent increase in cytosolic HMGB2 with increasing doses of CT-HMGB2 in both A549 NSCLC cells (EC_50_ = 3.4 nM, Fig. 8b) and HCT116 colon cancer cells (EC_50_ = 3.1 nM, Fig. 9a). The CT-HMGB2 also caused a dose dependent increase in translocation of CRT from the cytosol to the plasma membrane in both A549 NSCLC cells (EC_50_ = 12.4 nM, Fig. 8c) and HCT116 colon cancer cells (EC_50_ = 4.0 nM, Fig. 9b). In control experiments, recombinant (R)-HMGB1 and R-HMGB2 primarily remained in the media of both the A549 and HCT116 cells and none was found intracellularly. However, using IP to isolate the cell membranes, R-HMGB2 was found induce CRT translocation from the cytosol to the membranes of both A549 cells (47.1% of CT-HMGB2; Fig. 8d) and HCT116 cells (41.1 % of CT-HMGB2; Fig. 9c). R-HMGB1 did not cause any CRT translocation to the membrane of either A549 cells (Fig. 8d) or HCT116 cells (Fig. 9c). As was observed with oxaliplatin, the green fluorescence signal for CRT, after incubations of HCT116 colon cancer cells with oxaliplatin, CT-HMGB2 was present almost exclusively on the plasma membrane surface and there was a significant amount present after incubations with R-HMGB2 (Fig. 9d). Merging the blue Hoechst and green CRT signals showed that no CRT had translocated to the nucleus after incubations with oxaliplatin, CT-HMGB2 or R-HMGB2 (Fig. 9d). As was observed by western blot (Fig. 9c) no CRT translocation to the membrane surface was observed with R-HMGB1 (Fig. 9d). Oxaliplatin (100 mM) and R-HMGB2 (90 nM) were less efficient than CT-HMGB2 (90 nM) at inducing CRT translocation to the plasma membrane surface (Fig. 9d).

### Inhibition of XPO1-mediated oxaliplatin-induced HMGB nuclear export increases HCT116 decreases cell death

t has been reported previously that cytosolic HMGB1 and HMGB2 can regulate apoptosis through the activation of NF-kB^30,31^ and down regulation of Nrf2^32,33^, suggesting that inhibition of HMGB1 and HMGB2 secretion from the nucleus might ameliorate the oxaliplatin-mediated increase in cell death of HCT116 by preventing the down-regulation of Nrf2, a master controller of both ROS and lipid hydroperoxide detoxification pathways^32,33^ required to prevent ferroptosis^34^. Inhibition of the nuclear exporter XPO1 with 75 nM Selinexor, did indeed significantly reduce oxaliplatin-mediated HMGB1 (Fig. 5c) and HMGB2 secretion (Fig. 5d) and increased nuclear HMGB1 (Fig. 5g) and HMGB2 (Fig. 5h), which decreased cell death by 49.4 % from 34.4 ± 3.6 % to 17.4 ± 1.4 % (n = 5, p < 0.0006) when compared with oxaliplatin alone (Fig. 9e). Selinexor (75 nM) alone had no effect on cell viability or cell death (Fig. 9e). Ferrostatin-1 (10 mM), an inhibitor of ROS-induced ferroptotic cell death at this concentration^35^, reduced oxaliplatin-mediated cell death by 27.9 % to 24.8 ± 2.8 % (n = 5, p < 0.002).

## Discussion

ICD was characterized by Tesniere *et al*. as a cell death pathway relevant to certain chemotherapeutic agents that requires the release of soluble immunogenic signals including HMGB1 ^36^. Subsequently, numerous studies have implicated HMGB1 as an immunomodulatory DAMP because it can activate toll-like receptors (TLRs) including TLR2^37^, TLR4^38^, TLR9^39^ and/or the receptor for advanced glycation end products (RAGE)^40^ by well-characterized amino acid domains on the protein (Fig. 1b).^41,42^ We have now made the surprising observation that oxaliplatin causes the secretion of similar amounts of HMGB1 and HMGB2 from both A549 NSCLC and HCT116 colon cancer cells (Fig. 2); whereas cisplatin only causes HMGB1 secretion^3^. In contrast to the enormous literature on HMGB1 secretion,^43^ only a limited number of studies have examined the secretion of HMGB2^19^. Consequently, HMGB2 is not currently considered to be a DAMP^7^, even though (like HMGB1) it can activate RAGE^44,45^. There are few reported studies showing that HMGB2 can activate TLRs, although this activity is likely because HMGB2 and HMGB1 have identical amino acid sequences in the TLR4 binding region (amino acids 89–108, Fig. 1b).

Oxaliplatin-treated tumor cells are very effective at eliciting ICD, whereas tumor cells treated with other DNA-damaging platinum agents such as cisplatin and carboplatin are not^46^ (Fig. 1a). HMGB1 is not a primary mediator of ICD because cisplatin induced secretion of HMGB1 from cancer cells, does not cause cell death by this route^2^. On the other hand, oxaliplatin induces the rapid, pre-apoptotic translocation of CRT to the plasma membrane cell surface, an important requirement for ICD^2,46^. Interestingly, cisplatin can also cause translocation of CRT to the plasma membrane, and ICD induction, but only in the context of endoplasmic reticulum (ER) stress as induced with thapsigargin or tunicamycin^47^. CRT translocation also results from treatment of tumor cells with other DNA damaging agents including anthracyclines, bleomycin, and teniposide^26^. When CRT translocates to the plasma membrane surface of tumor cells, it is detected by the CD91 receptor on antigen-presenting cells, and its recognition prompts phagocytosis^48^. Translocation of CRT from the ER at early time points results in phagocytosis by immature dendritic like-cells; whereas, at later time points, macrophage-like cells are involved^49^. The role of CRT in oxaliplatin-induced ICD has been firmly established by depleting CRT with small inhibitory RNA (siRNA), which eliminates the immunogenicity of oxaliplatin^50^. Immunogenicity can then be readily restored by adding recombinant CRT protein back to the plasma membrane surface^50^. Thus, translocation of CRT from the ER is a key determinant of anticancer immune responses, which has been exploited as a target mechanism for immunogenic chemotherapy^26,27,51^.

We recently established that cisplatin induced HMGB1 secretion is mediated by the nuclear pore exporter XPO1^3^ rather than the widely reported acetylation of lysine residues on nuclear localization signal (NLS) 1 and NLS2 (Fig. 1b; highlighted in red)^52^. Using Selinexor (KPT-330), a potent XPO1 inhibitor, we have now conclusively established that HMGB2 is also secreted by XPO1 in A549 NSCLC and HCT116 colon cancer cells. Western blot analysis (Figs. 7b and 7d), immunofluorescence (Figs. 8a and 8b), and flow cytometry analysis (Figs. 8c and 8d) revealed that Inhibition of XPO1 in lung cancer and colon cancer cells also prevented oxaliplatin mediated translocation of CRT to the plasma membrane surface of the two cancer cell lines. This suggested that oxaliplatin induced secretion of HMGB2 into the cytosol (rather than HMGB1) was responsible for the CRT translocation because both untreated cell lines had significant levels of HMGB1 in the cytosol (Figs. 3b and 4b) but little HMGB2 (Figs. 3d and 4d). In addition, cisplatin induced HMGB1 secretion from the nucleus to the cytosol did not cause CRT translocation. It is puzzling that HMGB2 was detected in the cytosol of A549 cells (Fig. 3d) but not HCT116 cells after oxaliplatin treatment (Fig. 4d) as it was clearly secreted from the HCT116 cell nuclei (Fig. 4c) into the media (Fig. 2f). Consequently, HMGB2 must have been present in the HCT116 cell cytosol at some stage. This suggests that by 24-h, the HMGB2 was degraded in the cytosol and/or secreted into the extracellular milieu after causing translocation of CRT to the plasma membrane of the HCT116 cells.

To test whether intracellular HMGB2 could induce the translocation of CRT from the ER, CT-HMGB2 (Fig. 8a) was incubated with the A549 and HCT116 cell lines. CT-HMGB2 has a TAT sequence at the amino terminus (Fig. 8a), which enables it to cross the plasma membrane into the cytosol^28^. A dipeptide VA linker to the HMGB2 protein (Fig. 8a) can then be cleaved by cytosolic cathepsin B,^29^ which is up regulated in A549 NSCLC cells and HCT116 colon cancer cells^53,54^. CT-HMGB2 was efficiently taken up by both NSCLC and colon cancer cell lines, so that at the end of 24-h, none of the protein was detected in the incubation media of both cell lines. However, there was a dose-dependent increase of HMGB2 in the cytosol of both cell lines (Figs. 8b and 9a). This contrasts with R-HMGB1 and R-HMGB2 without a TAT sequence, which remained in the media of A549 and HCT116 cells after 24-h.

CT-HMGB2-derived cytosolic HMGB2 caused the translocation of CRT from the ER to the plasma membrane surface of both cell lines (Figs. 9c and 9g) in a similar manner to that observed for oxaliplatin (Figs. 7b and 7d). Confocal microscopy revealed that CT-HMGB2 caused complete translocation of CRT to the plasma membrane surface of HCT116 cells (Fig. 9d). The ratio of plasma membrane CRT to E-cadherin was 1.2 in A549 cells (Fig. 8c) and `1 in HCT116 cells (Fig. 9b), with a mean ratio of 1.2, after incubating the cells with 30 nM CT-HMGB2. The ratio of plasma membrane CRT to E-cadherin was 1.5 in A549 cells (Fig. 6b) and 1.1 in HCT116 cells (Fig. 6d), with a mean ratio of 1.3, after similar incubations with 100 μM oxaliplatin. This means that, CT-HMGB2 is > three orders of magnitude more potent than oxaliplatin. Surprisingly, non-targeted R-HMGB2 also caused the translocation of CRT to the plasma membrane surface of HCT116 cells (Fig. 9d), although it was significantly less potent than CT-HMGB2 in both A549 cells (Fig. 8d) and HCT116 cells (Fig. 9c). As might be predicted, R-HMGB1 did not cause translocation of CRT to the plasma membrane surface of A549 cells (Fig. 8d) or HCT116 cells (Figs. 9c and 9d). This raises the interesting possibility that HMGB2 can activate an extracellular receptor (that cannot be activated by HMGB1) which induces CRT translocation from the ER to the plasma membrane.

Oxaliplatin induces apoptosis in A549 cells^55^ *in vitro*; whereas oxaliplatin induces both apoptosis^56^ and ferroptosis in HCT116 cells^57–61^. Ferroptosis is a non-apoptotic type of iron-dependent programmed cell death, involving dysregulation of iron homeostasis and lipid peroxidation^62^. Cytosolic HMGB2 deficiency causes a decrease in angiotensin II-mediated ferroptosis and inflammation together with an increase in vascular smooth muscle cell viability by inactivating the NF-κB pathway^31^, which normally downregulates Nrf2^32,63^ and the expression of antioxidant enzymes^60^. Interestingly, oxaliplatin-induced ferroptosis^57^ was reduced (Fig 9e) when secretion of HMGB1 (Fig 4a) and HMGB2 (Fig. 4c) from the nucleus were inhibited by Selinexor. Although no HMGB2 was detected in the cytosol at 24-h in the absence of Selinexor (Fig. 4d). However, it was clearly present in the cytosol before it was secreted into the extracellular milieu. HMGB1 and HMGB2 are known to activate RAGE^45^, and so it is possible that the secreted HMGB1 and HMGB2 can also activate NF-κB^30,31^. Inhibition of oxaliplatin-mediated secretion of HMGB1 (Fig. 5g) and HMGB2 (Fig 5h) from the nucleus of HCT116 of cells by Selinexor would prevent NF-kB-mediated down regulation of Nrf2^30,31^, and so inhibit both ROS- and lipid hydroperoxide-mediated ferroptosis^34^. This explains why the reduction of HCT116 cell death by 49.4 % when compared with oxaliplatin alone, was greater than the 24.8 % reduction in cell death observed by inhibition of ROS-mediated ferroptosis with ferrostatin-1^62^ (Fig. 9e).

Inhibition of HMGB1 and HMGB2 secretion and/or preventing their activation of the NF-kB pathway^30,31^, could potentially prevent ferroptotic cell death in vascular smooth muscle cells^31^ and neurons^35^ *in vivo*. This offers a potential therapeutic approach to preventing ferroptotic cell death in cardiovascular^31^ and pulmonary diseases^64^ as well as neurodegenerative diseases^33^ such as Friedreich ataxia^65^. Intriguingly, inhibition of HMGB1 secretion^66^ and ferroptosis^35^ could also potentially prevent the progression of Alzheimer disease, where these pathways are thought to play important roles^33^. These data suggest that both HMGB1 and HMGB2 are mediators of ferroptosis; whereas HMGB2 alone can initiate CRT translocation (Fig. 9d).

It is noteworthy that immune checkpoint inhibitor (ICI) therapy appears to synergize with oxaliplatin-based, but not cisplatin-based cancer chemotherapy^67^. This has led to the suggestion that oxaliplatin and other chemotherapeutic agents that induce CRT translocation, could improve the efficacy of immunotherapies for immune-resistant “cold” tumors^27^. The improved potency of CT-HMGB2 for inducing CRT translocation in A549 NSCLC cells (EC_50_ = 12.4 nM) and HCT116 colon cancer cells (EC_50_ = 4.0 nM), when compared with oxaliplatin, means that it could be a very useful adjunct to immunotherapy. In addition, Neubert *et al*. recently discovered a previously unknown role for HMGB2 in the differentiation and survival of functional memory cells as well as stem-like progenitor exhausted T cells^20^. Nuclear HMGB2 makes an important contribution to T-cell factor-1 (TCF-1) and thymocyte selection-associated HMG box (TOX) mediated regulation of T-cell exhaustion through its ability to induce chromatin remodeling. The mechanism of action of ICIs requires the correction of T cell exhaustion, which might explain why ICIs are only effective in a minority of cancer patients with “hot” tumors^68^. Targeting HMGB2 might enable “cold” tumors to become responsive to ICI therapy by two distinct and complementary mechanisms: inducing ICD (Fig. 9d), and modulating T cell exhaustion^20^. Therefore, targeted protein therapy with novel proteins like CT-HMGB2 could complement the current armamentarium of therapies used in the treatment of cancer as well as expanding the proportion of patients responsive to immune based therapies.

## Methods

For a complete list of antibodies, reagents, and materials see Supplementary Table 1

### HMGB proteins

Stable isotope labeling by amino acids in cell culture (SILAC)-HMGB1 was prepared as described previously^3^. Gene fragments for R-HMGB1, R-HMGB2, or CT-HMGB1 were subcloned into a pET-30a (+) vector with an N-terminal 6xHis tag by GenScript (Piscataway, NJ). The plasmid was synthesized, and the relevant HMGB proteins expressed by the GenScript service. The plasmid was transformed into BL21 Star (DE3) strain, where it underwent overnight growth on a kanamycin-resistant (K+) solid culture medium plate. For the preparation of a glycerol stock strain, a single colony was picked and inoculated into 4–5 mL of Luria-Bertani (LB) culture medium (K+). Subsequently, a 50 mL seed culture was prepared and then inoculated into TB medium at a 1:100 ratio. Cells were grown at 37°C until reaching an OD600 value of 1.2, followed by induction of expression using 0.5 mM isopropyl β-d-1-thiogalactopyranoside (IPTG), and expressed at 15 °C for 16-h. Cells were harvested by centrifugation and stored at −80°C. Cell pellets were resuspended with lysis buffer (50 mM Tris-HCl, 150 mM NaCl, pH 8.0) followed by sonication. The supernatant after centrifugation was purified by Ni column (GenScript NTA-Ni) affinity chromatography. Protein fractions were finally dialyzed into imidazole-free buffer (50 mM Tris-HCl, 150 mM NaCl, 10 % Glycerol, pH 8.0) and HMGB proteins stored at −80°C until used.

### Cell culture and intervention

HCT116 colon cancer cells were cultured in McCoy’s medium and A549 NSCLC cells were cultured in Dulbecco′s modified Eagle′s medium (DMEM) each supplemented with 10 % fetal bovine serum (FBS) and 1 % penicillin under 5 % CO_2_ at 37 °C. Oxaliplatin was dissolved and sonicated in the relevant culture medium for each type of cell. Selinexor was diluted to the working solution using the relevant culture medium. The final concentration of DMSO was ≤ 0.1%, which had no effect on the cell viability.

### Trypan blue and propidium iodide (PI) fluorescence staining

Cells (1−1.5 × 10^6^) were trypsinized in 10 mL of media using 0.25 % trypsin solution (4 mL) for 5-min at 37 °C to prepare a cell suspension. The cell suspension was mixed with 0.4 % Trypan Blue solution in a 1:1 ratio and vortex-mixed for 2-min at room temperature. The blue stained dead cells were counted within 3-min using a Luna-FL automated fluorescence cell (Logos Biosystems, Annandale, VA). Dead cells were stained with a clear blue color, while live cells were colorless and transparent, which were used to determine dead cell count, cell viability and total cell count. A working solution of calcein AM stain (2 μM) was prepared in PBS with ≤ 0.1% DMSO and enough of the solution was added to adequately cover the adherent cells. Cells in suspension were pelleted by centrifugation, washed once in Hank’s balanced salt solution (HBSS), and incubated for 30-min at 37 °C. Each sample was equilibrated briefly in 2X saline sodium citrate (SSC) buffer (0.3 M NaCl, 0.03 M sodium citrate, pH 7.0). Cell samples was incubated in 100 μg/mL DNase free RNase dissolved in 2X SSC for 20-min at 37°C. A 500 nM solution of PI was prepared in 2X SSC, and the cells were covered with 300 μL of the PI solution for 5-min. The green calcein stained live cells and the red PI stained apoptotic and necrotic cells were counted using a Leica DM750 HD digital fluorescence microscope (Leica Microsystems Inc., Deerfield, IL).

### Sub-cellular protein isolation

Cytosolic and nuclear proteins were isolated using a subcellular protein fractionation kit for cultured cells (# 78840). The protocol was adapted from that supplied with the kit. Cells (1 −1.5 × 10^6^) were trypsinized with 0.25 % trypsin solution (4 mL) and then centrifuged at 500×g for 5-min in a ST40R centrifuge (Thermo Scientific, Waltham, MA). Cells were washed by suspending the cell pellet in ice-cold PBS (1 mL). Proprietary ice-cold cytoplasmic extraction buffer (CEB, 1 mL) containing 1 μL protease inhibitors was added to the cell pellet, the pellet was incubated at 4°C for 10-min with gentle mixing, then centrifuged at 500×g for 5-min in a 5430R microcentrifuge (Eppendorf, Hauppauge, NY). The supernatant was removed and provided the cytoplasmic fraction for further analysis. Ice-cold nuclear extraction buffer (NEB, 1 mL) containing 1 μL of protease inhibitors was added to the pellet after the supernatant had been removed, and vortex-mixed at the highest setting for 15-sec. The NEB mixture was incubated at 4°C for 30-min with gentle mixing, then centrifuged at 5000×g for 5-min using a microcentrifuge . The supernatant provided the soluble nuclear fraction for further analysis. Cell plasma membranes were prepared with the Mem-PER Plus Kit (#89842). Cells (1 −1.5 × 10^6^) were suspended in growth media of the relevant cells by scraping the cells off the surface of the plate with a cell scraper. The harvested cell suspension was centrifuged at 300×g for 5-min in a 5430R microcentrifuge. The cell pellet was washed with the provided proprietary cell wash solution (3 mL) and centrifuged at 300×g for 5-min in the 5439R microcentrifuge. The provided proprietary permeabilization buffer (0.75 mL) was added to the cell pellet and incubated for 30-min at 4 °C with constant mixing. Permeabilized cells were centrifuged for 15-min at 16,000×g in the 5409R microcentrifuge. The supernatant was removed and provided the cytoplasmic portion for further analysis. Proprietary membrane solubilization buffer (0.5 mL) that was provided in the kit was added to the pellet after removal of the supernatant and membranes re-suspended by pipetting up and down. After incubating at 4°C for 50-min with constant mixing, the membrane preparation was centrifuged at 16,000×g for 15-min at 4°C in the microcentrifuge. Separation of the supernatant provided the plasma membrane fraction for further analysis.

### Western blot analysis of media

Cell media (200 mL) from HCT116 or A549 cells was concentrated with nitrogen gas using an N-Evap concentrator (Organomation, West Berlin, MA). The residue was dissolved in 20 mL of Nupage sample loading buffer, which was loaded on a 10% NuPAGE Bis-Tris protein gel. The gel was run under 150 V for 1.5 h until the blue dye ran to the bottom of the gel. The proteins were transferred to an Invitrogen nitrocellulose membrane and HMGB1 detected by incubation overnight at 4 °C with an HMGB1 rabbit pAb (ab79823) primary antibody. An incubation with a secondary anti-rabbit HRP secondary antibody was then conducted for 1.5-h. The blots were developed with the electrochemical luminescence (ECL) reagent (Revvity) and visualized with an ImageQuant LAS 4000 camera (GE Healthcare, Piscataway, NJ). HMGB2 was similarly detected via a primary HMGB2 rabbit pAb (ab124670) and a secondary anti-rabbit HRP antibody. Western blots for HMGB1 and HMGB2 were then quantified using open-source Image J software^69^. The membrane western blot signals were normalized by subtraction of the membrane blank at the appropriate gel mobility, and the grayscale value determined for each blot.

### IP of cell membrane CRT for western blot analysis

Protein A/G magnetic beads (40 μL) were transferred to 2-mL Eppendorf protein LoBind tubes. The beads were washed twice with DPBS and twice with buffer A (0.1 M sodium phosphate, pH 7.4). The tubes were then incubated at 4 °C overnight with buffer A (500 μL) and rabbit anti-CRT pAb (50 μL; ab227444). Rabbit anti-CRT pAb solution was removed and beads were washed gently with 1 mL of cross-linking buffer (0.2 M triethanolamine, pH 8). The beads were then suspended in 1 mL of 25 mM dimethylpimelimidate (DMP) prepared in cross-linking buffer and incubated at room temperature for 1-h with gentle rotation. The DMP solution was removed, and the beads were washed with 1 mL of blocking buffer (0.1 M ethanolamine, pH 8.2) and incubated at room temperature for 30 min in 1 mL of blocking buffer. The beads were then incubated in elution buffer (0.1 M glycine-HCl) for 15 min at room temperature with gentle rotation. After removing the elution buffer, covalently bound CRT pAb beads (10 mL) were aliquoted into Eppendorf protein LoBind tubes containing 1 mL of HCT116 or 1 mL of A549 cell suspension (1−1.5 × 10^5^). Intact cells were incubated for 6-h at 4 °C with gentle rotation to allow the plasma membrane CRT to bind to the covalently bound CRT pAb. Cells were then discarded, and the beads were washed 2 x with DPBS (1 mL). The beads were shaken vigorously in 100 μL elution buffer for 10-min and then for a further 5 min with 10 mM NH_4_HCO_3_ (50 μL). The beads were removed, which left the CRT protein in the supernatant. Each sample was then neutralized with 250 mM NH_4_HCO_3_ (50 μL) and centrifuged 7,000×g for 10 min in a 5430R microcentrifuge to remove any residual cell debris. Loading buffer was added (50 mL) and 15 mL of the solution containing CRT protein analyzed by western blot as described for the subcellular fractions.

### Western blot analysis of subcellular fractions

The residue from nuclear, cytoplasmic, and plasma membrane samples was dissolved in 10 mL of Nupage sample loading buffer. Total protein concentration was quantified using the BCA protein assay to ensure equal amounts of protein were loaded on the gel for different sample groups. Typically, 4 mg of total protein from each of the sub-cellular fractions was loaded on a 10% NuPAGE Bis-Tris protein gel. The gel was run under 150 V for 1.5 h until the blue dye ran to the bottom of the gel. After PAGE separation was completed, proteins were transferred to a nitrocellulose membrane, which was then incubated overnight at 4 °C with the primary antibody. An incubation with a secondary antibody at room temperature for 1.5-h was then performed. The blots were developed with the ECL reagent and visualized with an ImageQuant LAS 4000 camera (GE Healthcare, Piscataway, NJ). Blots quantified with Image J^69^ as described above. The primary and secondary antibodies for HMGB1 and HMGB2 were the same as for the media samples described above. The primary antibody for CRT was rabbit anti-CRT monoclonal antibody (mAb; ab22744), for fatty acid synthase (FASN) was rabbit anti-FASN mAb (MA5–14887), for histone H4 was rabbit anti-H4 mAb (16047-IAP), ), and for E-cadherin was rabbit anti- E-cadherin mAb (EP700Y). The secondary antibody for CRT, FASN, histone H4, and E-cadherin was goat anti-Rabbit IgG (#7074S). To add molecular weigh information to the western blots, digital photograph of the membrane was taken by the ImageQuant LAS 4000 camera immediately after chemiluminescent imaging. The image was them temporarily overlayed in Photoshop to manually mark the molecular weight markers on the chemiluminescent image.

### Flow cytometry

HCT116 (1−1.5 × 10^5^) cells and A549 cells (1−1.5 × 10^5^) were harvested and washed twice with PBS. The cells were then incubated for 1.5 h in the dark at 4 °C with PE-Cy-7-labeled anti-CRT that had been labeled with the Abcam PE/Cy7 conjugation kit following the manufacturer’s instructions. HCT116 and A549 cells were then re-suspended in cold PBS (1.5 mL) for analysis on the NovoCyte Advanteon flow cytometer system (Agilent Technologies, Santa Carla, US). Natural cell fluorescence was monitored by excitation at 564 nm and emission at 606 nm; whereas CRT fluorescence was monitored by excitation at 496 nm and emission at 774 nm. Results from the flow cytometer were analyzed with BD FACSuite Software (BD Biosciences, San Jose, CA).

### Immunofluorescence

HCT116 cells (1−1.5 × 10^4^) cells or A549 cells (1−1.5 × 10^4^) were deposited on a chamber slide (#177380) of a confocal microscope. Cells were washed with 1 mL of PBS and fixed at room temperature with 4% paraformaldehyde for 10-min. The fixed cells were incubated with 500 μL of 0.1% Triton for 5-min to permeabilize the cell membrane by creating holes on their cell surface. Cells were then incubated with 500 μL of goat serum at room temperature for 1-h, followed by an incubation with a primary mouse anti-CRT mAb (ab22683) for 20-h at 4 °C, and a room temperature incubation with a green fluorescent labeled secondary antibody (goat anti-mouse IgG H&L; Alexa Fluor^®^ 488) for 1-h. Cell permeable Hoechst 3342 dye was used to visualize the presence of nuclear DNA in the cells by staining for 15-min. Hoechst 3342 binds to the minor groove of DNA at A-T-rich regions and emits blue fluorescence when bound to double-stranded DNA. Images were acquired with an Olympus FV1000 confocal microscope (Evident Scientific, Waltham MA ) at 1000 × magnification.

### Gel protein digestion for nano LC-PRM/HRMS analysis

Media (200 mL) from HCT116 or A549 cells was spiked with SILAC-HMGB1, concentrated with nitrogen gas using an N-Evap concentrator (Organomation), and dissolved in 10 mL Nupage sample loading buffer (10 mL). Samples were loaded on a 10% NuPAGE Bis-Tris protein gel. The gel was run under 150 V for 1.5-h until the blue dye ran to the bottom of the gel. Bands corresponding to a mobility of between 25 kDa and 37 kDa were removed with a surgical blade, sliced into 1 mm^3^ gel pieces, and de-stained by rinsing these pieces twice in 25 mM NH_4_HCO_3_ buffer/50% acetonitrile solution. The gel pieces were then dehydrated in 100 % acetonitrile, vortex-mixed for 10 min, and the supernatant was discarded. They were then suspended in 25 mM NH_4_HCO_3_ (100 μL) and vortex-mixed for 10-min at room temperature. Chymotrypsin (500 ng) in buffer (100 mM Tris HCl and 10 mM CaCl_2_; 50 μL) was added to each sample, the pH was adjusted to 8.0, and incubations were conducted for 20-h at room temperature. After the digestion, the supernatant was transferred to a clean 1.5 mL protein LoBind tube, and 200 μL of extraction buffer (3% formic acid in 50% aqueous acetonitrile) was added to the gel pieces. The mixture was sonicated at 37 °C for 30 min. The supernatant was dried under N_2_, water (50 μL) was added, and the solution was transferred to deactivated glass inserts ready for LC-MS analysis.

### Nano-LC-PRM/HRMS

Analysis was carried out using a Q Exactive HF hybrid quadrupole-Orbitrap mass spectrometer coupled to a Dionex Ultimate 3000 RSLCnano with a capillary flowmeter chromatographic system, supplied by Thermo Fisher Scientific (San Jose, CA, USA). The nano-LC system comprised a trapping column (Acclaim PepMap C18 cartridge, 0.3 mm×5 mm, 100 Å, Thermo Scientific) for preconcentration, and an analytical column (C18 AQ nano-LC column with a 10 μm pulled tip, 75 μm × 25 cm, 3 μm particle size; Columntip, New Haven, CT, USA) for peptide separation. The nano-LC system included two pumps: a nanopump delivering solvents to the analytical column and a micropump connected to the trapping column. Additionally, a 10-port valve was part of the system. Xcalibur software was used to control the nano-LC system. Samples (8 μL) were injected using the microliter-pickup injection mode. The loading solvent, composed of water/acetonitrile (99.7:0.3 v/v) with 0.2 % formic acid, was used at a rate of 10 μL/min for 3 min. During the analysis, the 10-port valve was initially set at the loading position (1–2) with the loading solvent, and after 3 min, it switched to the analysis position (1–10). At this point, the trapping column was connected to the analytical column, and the loaded samples were back flushed into the analytical column. The valve remained in the analysis position for 10-min before returning to the loading position for the next analysis. A linear gradient elution was employed at a flow rate of 0.35 μL/min, starting with 2 % B at 2 min, reaching 5 % B at 15 min, 35 % B at 40 min, 95 % B at 45–55 min, and returning to 2 % B at 58–70 min. Solvent A was water/acetonitrile (99.5:0.5 v/v) with 0.1% formic acid, while solvent B was acetonitrile/water (98:2 v/v) with 0.1 % formic acid. A Nanospray Flex ion source (Thermo Scientific) was utilized. MS operating conditions were set as follows: spray voltage 2500 V, ion transfer capillary temperature 250 °C, positive ion polarity, S-lens RF level 55, in-source collision-induced dissociation (CID) 1.0 eV. Both full-scan and parallel reaction monitoring (PRM) modes were employed. Full-scan parameters included a resolution of 60,000, automatic gain control (AGC) target of 1×10^6^, maximum IT of 200 ms, and a scan range of m/z 290–1600. PRM parameters involved a resolution of 60,000, AGC target of 2 × 10^5^, maximum IT of 80 ms, loop count of 5, isolation window of 1.0 Da, and normalized collision energy (NCE) of 25.

### Quantification of HMGB1 and HMGB2

Standard curve samples for tryptic of HMGB1 and HMGB2 were prepared in media in triplicate in the range 65 to 680 ng. The HMGB1 tryptic peptide K^90^DPNAPKRPPSAF^102^, was used to quantify HMGB1 in A549 and HCT116 media with **K**^90^DPNAP**K**RPPSAF^102^ as the internal standard (**K** = [^13^C_6_^15^N_2_-]-lysine). There is no equivalent chymotryptic peptide from HMGB2 because F-89 is replaced by K-89 in HMGB2 (Figure 1a). Tryptic peptide S^134^EQSAKDKQPY^144^, which has five amino acid differences compared with the corresponding HMGB1 tryptic peptide N^134^NTAADDKQPY^144^, was used to quantify HMGB2 in A549 and HCT116 media. Standard curves for HMGB2 were prepared using S^134^EQSAKDKQPY^144^ peptide intensities with no internal standard (Supplementary Table 2). Light to heavy peptide ratios were calculated from the sum of the three most intense PRM transitions for the HMGB1 tryptic peptide K^90^DPNAPKRPPSAF^102^ or the signal intensity from the sum of three most intense transitions (x10^−6^) for the HMGB2 tryptic peptide S^134^EQSAKDKQPY^144^ (Supplementary Table 2). A typical regression line for K^90^DPNAPKRPPSAF^102^ was y = 0.01306x - 0.3176 (r^2^ = 0.9856) and for S^134^EQSAKDKQPY^144^ was y = 0.1594x - 1.015 (r^2^ = 0.9989). Back calculated values for the standards were with 85 % and 115 % of the theoretical values. Interpolation of light to heavy peptide ratios for the HMGB1 peptide or the sum of the three most intense transitions for the HMGB2 peptide in the relevant linear standard curve then provided the amount of HMGB1 or HMGB2 in the cell media.

### Data analysis

Data comparisons between two different groups were performed using the Student’s unpaired two tailed t-test and EC_50_ values were calculated using GraphPad Prism (Prism 10 for Mac Version 10.1.1, November 21, 2023). A p value of < 0.05 was regarded as statistically significant. All error bars are expressed as ± standard deviation (SD) Processing of mass spectrometry data and standard curves was conducted using Skyline software (MacCoss Laboratory, University of Washington, Seattle, WA)^70^.

## Data Availability

The data that support the findings of this study are available within the paper. Full size western blots, numerical source data underlying graph plots in the manuscript, and figures exemplifying the gating strategy can be found in Supplementary data 3, Any additional information not included in the paper is available upon request from Dr. Ian A. Blair.
